# Southern and Western Convention at Memphis

**Published:** 1845-11

**Authors:** 


### SOUTHERN AND WESTERN CONVENTION AT MEMPHIS.

We have lately received a circular, giving extracts from the proceedings of a popular convention held at Memphis, Tennessee, on the 4th and 5th days of July last, for the promotion of Western and Southern interests, among which is the establishment of Marine or Commercial Hospitals on the western rivers. It is this subject which entitles the convention to a notice in our journal. The committee to whom it was confided, consists of Drs. Cartright of Natchez, Stone of New Orleans, Balfur of Vicksburg, W. Christian of Memphis, H. Lane of St. Louis, Drake of Louisville, Rives of Cincinnati, Houston of Wheeling, and Irwin of Pittsburgh. We have lately received a letter from our indefatigable friend, Dr. Shanks, of Memphis, (who, by the way, was president of the convention), in which he expresses the apprehension, that as the members of the committee are scattered along the river, for 2000 miles, there will not be a meeting, nor any report prepared for the next Convention, to be held on the 14th instant. Should no report be forthcoming at that time, the blame must rest on the chairman of the committee, Dr. Cartright, who is so eminently qualified for his station, that he will find it difficult to invent an excuse, for not having corresponded with the various members of the committee, and digested their facts and opinions into a regular report. It is now 10 or 12 years since the necessity of a national hospitals on the Mississippi, Ohio, and the Lakes, was, on the original suggestion of Dr. Campbell, of St. Louis, urgently presented to the consideration of Congress; and sites were purchased at a number of places, but the erection of hospitals on most of them was not undertaken, and the few that were commenced, were suspended before they were finished. It is 'high time,' that the government was stimulated to proceed in this matter, and we hope our readers will do what they can with their members of Congress to rouse them into action. D.

